# *Plesiomonas shigelloides* Infection, Ecuador, 2004–2008

**DOI:** 10.3201/eid1802.110562

**Published:** 2012-02

**Authors:** Juan C. Escobar, Darlene Bhavnani, Gabriel Trueba, Karina Ponce, William Cevallos, Joseph Eisenberg

**Affiliations:** University of Michigan, Ann Arbor, Michigan, USA (J. Eisenberg, D. Bhavnani);; Universidad San Francisco de Quito, Quito, Ecuador (G. Trueba, W. Cevallos, K. Ponce, J.-C. Escobar)

**Keywords:** Plesiomonas, diarrhea, Ecuador, co-infection, risk, pathogenicity, bacteria

## Abstract

Diarrheal risk associated with *Plesiomonas shigelloides* infection was assessed in rural communities in northwestern Ecuador during 2004–2008. We found little evidence that single infection with *P. shigelloides* is associated with diarrhea but stronger evidence that co-infection with rotavirus causes diarrhea.

*Plesiomonas shigelloides* (family Enterobacteriaceae) has been implicated in gastroenteritis outbreaks in travelers to tropical regions and in persons who have ingested contaminated food or water (*1**–**3*). For persons native to tropical regions, however, case–control studies have found little or no association between *P. shigelloides* infection and diarrhea (*4**–**6*). Although these studies have been conducted in areas where mixed infections are generally common, to our knowledge, none examined co-infections. We assessed the pathogenicity of *P. shigelloides* in the context of co-infections and across all age groups in a province in northwestern Ecuador.

## The Study

During 2004–2008, serial case–control studies were conducted in 22 remote communities in Esmeraldas Province, Ecuador. Complete study design and laboratory procedures for pathogen detection have been described (*7*). Briefly, each community was visited 4–6 times on a rotating basis; each visit lasted for 15 days, during which all cases of diarrhea were identified by a visit to each household every morning. Household residents with cases had >3 loose stools in a 24-hour period, and controls had no symptoms of diarrhea during the past 6 days. Fecal samples were collected from 3 healthy controls per person with diarrhea. These samples were plated on selective agar media, and 5 lactose-fermenting colonies were screened by PCR for enterotoxigenic *Escherichia coli* (ETEC), enteropathogenic *E. coli*, and enteroinvasive *E. coli* (EIEC). Lactose-negative isolates that were identified as either *Shigella* spp. or *E. coli* were also screened by PCR for the same molecular marker used for EIEC. All non–lactose-fermenting pathogens, including *P. shigelloides*, were biochemically identified by API 20E system (bioMèrieux, Marey l’Etoile, France). Because shigellae are phylogenetically similar to *E. coli* pathotypes, we combined data from persons infected with *E. coli* and those infected with shigellae in our analysis. We tested for *Giardia lamblia* by using an ELISA kit (RIDASCREEN *Giardia*; R-Biopharm, Darmstadt, Germany), and rotavirus was detected with an enzyme immunoassay kit (RIDA Quick Rotavirus; R-Biopharm). We chose a molecular method for detecting *E. coli* pathotypes because they cannot be differentiated solely on the basis of biochemical tests; the metabolic homogeneity of *P. shigelloides*, however, makes this organism easily and clearly identifiable by biochemical test. Similarly, immunologic methods used for *Giardia* spp. and rotavirus detection are specific and sensitive enough to accurately detect these pathogens, and use of molecular methods would be justified only for deeper analysis. Institutional review board committees at the University of California, Berkeley; University of Michigan; Trinity College; and Universidad San Francisco de Quito approved all protocols.

During March 2004–March 2008, a total of 2,936 fecal samples were collected from persons of all ages (168 [6%] were <1 year of age, 597 [20%] were 1–4 years, 753 [26%] were 5–12 years, 1,362 [46%] were >13 years, and 56 [2%] were missing a birth date), corresponding to 775 cases and 2,161 controls. *P. shigelloides* was isolated in 253 (8.6%) samples. This number exceeded isolation rates for all of the pathogens analyzed except *G. lamblia*, which was present in 701 (23.9%) samples. Rotavirus was detected in 225 (7.7%) samples and EIEC/shigellae in 188 (6.4%) samples. *P. shigelloides* was detected in 11.4% of case-patients with diarrhea (case prevalence), which is more than the 7.2% estimated in the community (weighted control prevalence; Figure). However, once we stratified by persons infected only with *P. shigelloides* and those infected with *P. shigelloides* plus >1 of the other marker pathogens for which we tested, single infections with *P. shigelloides* were almost equally prevalent in the case-patients and in the community; in contrast, co-infections with *P. shigelloides* and other pathogens were more frequent in persons with diarrhea (Figure).

**Figure:**
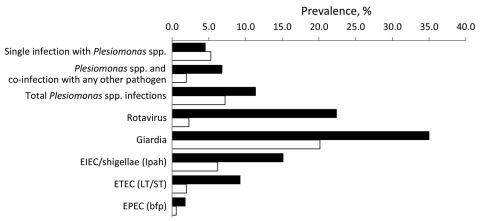
Case prevalence (black) and weighted community prevalence (white) of enteric pathogens, Ecuador, 2004–2008. Identification of pathogenic *Escherichia coli* was based on the genes given in parentheses. EIEC, enteroinvasive *E. coli*; Ipah, invasion plasmid antigen gene; ETEC, enterotoxigenic *E. coli*; LT, heat-labile toxin; ST, heat-stable toxin; EPEC, enteropathogenic *E. coli*; bfp, bundle-forming pili.

To determine whether *P. shigelloides* infection was associated with diarrhea, we estimated risk ratios (RRs) and bootstrapped 95% CIs for single and co-infection exposures (Table). A single infection with *P. shigelloides* was not associated with diarrhea (RR 1.5, 95% CI 0.9–2.2). Persons co-infected with *P. shigelloides* and another pathogen, however, had almost 6× the risk for diarrhea than those with no infection (RR 5.6, 95% CI 3.5–9.3) and simultaneous occurrence of *P. shigelloides* and rotavirus increased the risk for diarrhea to 16.2 (Table). We found no evidence for confounding of the association between *P. shigelloides* and diarrhea by co-infecting pathogens (RR_crude_ = RR_MH-pooled_; where RR_crude_ is the unadjusted RR and RR_MH-pooled_ is the pooled Mantel-Haenszel RR estimate). However, we found some evidence for confounding by age of *P. shigelloides* co-infection (RR_MH-pooled_ 4.2 [95% CI 2.1–8.1], compared with the crude estimate of 5.6) but no evidence for confounding by age for single infection with *P. shigelloides*.

**Table:** RRs and bootstrapped 95% CIs for single infections and co-infections with *Plesiomonas shigelloides*, Ecuador, 2004–2008*

Co-infection	RR_Single P.shig_ (95% CI)	RR_Co-Infection_ (95% CI)	RR_Crude_ (95% CI)	RR_MH-Pooled_ (95% CI)	Wald test for heterogeneity	p value
Any pathogen	1.5 (0.9–2.2)	5.6 (3.5–9.3)	2.6 (1.9–3.5)	2.7 (1.9–3.6)	32.1	<0.001
Rotavirus	1.5 (0.9–2.2)	16.2 (5.5–62.3)	1.7 (1.1–2.5)	1.9 (1.2–2.9)	61.8	<0.001
*Giardia *spp.	1.5 (0.9–2.2)	2.1 (1.0–3.9)	1.5 (1.0–2.2)	1.6 (1.1–2.3)	1.3	0.2
*Escherichia coli*/ shigellae	1.5 (0.9–2.2)	13.8 (3.3–69.3)	1.6 (1.1–2.4)	1.7 (1.1–2.6)	32.8	<0.001

*RR, risk ratio. RR_crude_ = the unadjusted RR and RR_MH-pooled_ is the pooled Mantel-Haenszel RR ratio estimate. The Wald test assesses whether the strata RR_Single P.shig_ and RR_co-infection_ differ. Because of the clustered study design and the unequal sampling probabilities of controls, we chose not to use logistic regression models. Instead, we applied a nonparametric approach by using sampling weights to estimate RRs, as one would for a cohort study. We bootstrapped 1,000 samples from the original dataset, and with each new sample, we estimated the RR associated with single infection and co-infection. The lower 0.025 and upper 0.975 percentiles of the bootstrap distribution are reported as 95% CIs. Statistical analyses were conducted by using R version 2.11.1 (www.r-project.org).

## Conclusions

A single infection with *P. shigelloides* resulted in a moderately increased risk for diarrheal disease, which suggests that this microorganism plays a minor role as a pathogen. This result agrees with findings of previous studies (*4**,**8**,**9*). Analysis of the co-infections, however, suggests that *P. shigelloides* may be pathogenic in the presence of another pathogen. Specifically, co-infections of *P. shigelloides* with either rotavirus or pathogenic *E. coli* (including shigellae) were 16.2× (95% CI 5.5–62.3) and 13.8× (95% CI 3.3–69.3) more likely to result in diarrhea, respectively. We cannot, therefore, rule out the pathogenic capacity of *P. shigelloides* even though single infection may not be sufficient to cause disease.

This co-infection analysis might be limited by the number of pathogens considered (*Giardia* spp., rotavirus, pathogenic *E. coli*, and shigellae). However, the high isolation rates suggest we are detecting the major pathogens in the region. Other pathogens that may be useful to consider, given their attention in the literature, include *Entaemobae histolytica* and *Cryptosporidium* spp.

Although we found nothing in the literature that addresses the role of co-infection in the pathogenicity of *P. shigelloides*, co-infection with enteric pathogens is a well-known phenomenon, especially in tropical regions (*6*). Co-infection with ETEC and enteropathogenic *E. coli* increases virulence (*10*). Other studies have shown that the severity of disease is increased when rotavirus infections occur alongside another infection with another enteric pathogen (*11*).

*P. shigelloides* may take advantage of the disruption of the normal gut microbiota and gut physiology because of the concurrent presence of other pathogens, establishing a pathology in the human gut. For example, diarrhea caused by enterotoxins produced by pathogens, such as ETEC, and *Vibrio cholerae* (*12*), may remove normal gut microbiota, enabling *P. shigelloides* to establish an infection. The disruption of gut microbiota that facilitates gut colonization has been demonstrated in murine models infected with *Citrobacter rodentium* and *Salmonella enterica* serovar Typhimurium (*13*).

Most medical literature considers infectious diarrhea as a monopathogenic phenomenon (*12**,**14*). In the data presented here, the crude risk ratio suggests that *P. shigelloides* is pathogenic. When looking at single infections, we found no evidence that *P. shigelloides* is pathogenic. When looking at co-infection data, however, we found associations between infection and diarrhea. Our findings suggest that multipathogenic infections may play a role in the pathogenesis of infectious diarrhea.

## References

[1] Adams MR, Moss MO. Food microbiology. 3rd ed. Cambridge (UK): The Royal Society of Chemistry; 2008.

[2] Brenden RA, Miller MA, Janda JM. Clinical disease spectrum and pathogenic factors associated with *Plesiomonas shigelloides* infections in humans. Rev Infect Dis. 1988;10:303–16. 10.1093/clinids/10.2.303 3287561

[3] Kain KC, Kelly MT. Clinical features, epidemiology, and treatment of *Plesiomonas shigelloides* diarrhea. J Clin Microbiol. 1989;27:998–1001. 2745707 10.1128/jcm.27.5.998-1001.1989 PMC267470

[4] Pitarangsi C, Echeverria P, Whitmire R, Tirapat C, Formal S, Dammin GJ, Enteropathogenicity of *Aeromonas hydrophila* and *Plesiomonas shigelloides*: prevalence among individuals with and without diarrhea in Thailand. Infect Immun. 1982;35:666–73. 7056580 10.1128/iai.35.2.666-673.1982 PMC351093

[5] Alabi SA, Odugbemi T. Occurrence of *Aeromona*s species and *Plesiomonas shigelloides* in patients with and without diarrhoea in Lagos, Nigeria. J Med Microbiol. 1990;32:45–8. 10.1099/00222615-32-1-45 2342086

[6] Bodhidatta L, McDaniel P, Sornsakrin S, Srijan A, Serichantalergs O, Mason CJ. Case–control study of diarrheal disease etiology in a remote rural area in western Thailand. Am J Trop Med Hyg. 2010;83:1106–9. 10.4269/ajtmh.2010.10-0367 21036846 PMC2963978

[7] Eisenberg JN, Cevallos W, Ponce K, Levy K, Bates SJ, Scott JC, Environmental change and infectious disease: how new roads affect the transmission of diarrheal pathogens in rural Ecuador. Proc Natl Acad Sci U S A. 2006;103:19460–5. 10.1073/pnas.0609431104 17158216 PMC1693477

[8] Abbott SL, Kokka RP, Janda JM. Laboratory investigations on the low pathogenic potential of *Plesiomonas shigelloides.* J Clin Microbiol. 1991;29:148–53. 1993749 10.1128/jcm.29.1.148-153.1991 PMC269719

[9] Albert MJ, Faruque AS, Faruque SM, Sack RB, Mahalanabis D. Case–control study of enteropathogens associated with childhood diarrhea in Dhaka, Bangladesh. J Clin Microbiol. 1999;37:3458–64. 10523534 10.1128/jcm.37.11.3458-3464.1999 PMC85667

[10] Crane JK, Choudhari SS, Naeher TM, Duffey ME. Mutual enhancement of virulence by enterotoxigenic and enteropathogenic *Escherichia coli.* Infect Immun. 2006;74:1505–15. 10.1128/IAI.74.3.1505-1515.2006 16495521 PMC1418639

[11] Grimprel E, Rodrigo C, Desselberger U. Rotavirus disease: impact of coinfections. Pediatr Infect Dis J. 2008;27:S3–10. 10.1097/INF.0b013e31815eedfa

[12] Sussman M, editor. Molecular medical microbiology. London: Academic Press; 2002.

[13] Viswanathan VK, Hodges K, Hecht G. Enteric infection meets intestinal function: how bacterial pathogens cause diarrhoea. Nat Rev Microbiol. 2009;7:110–9. 19116615 10.1038/nrmicro2053 PMC3326399

[14] Guandalini S, Vaziri H, eds. Diarrhea: diagnostic and therapeutic advances. London: Springer; 2011.

